# Recalibrating the Non-Communicable Diseases risk prediction tools for the rural population of Western India

**DOI:** 10.1186/s12889-022-12783-z

**Published:** 2022-02-22

**Authors:** Manoj Kumar Gupta, Pankaja Raghav, Tooba Tanvir, Vaishali Gautam, Amit Mehto, Yachana Choudhary, Ankit Mittal, Gyanendra Singh, Garima Singh, Pritish Baskaran, V.R. Rehana, Shaima Abdul Jabbar, S. Sridevi, Akhil Dhanesh Goel, Pankaj Bhardwaj, Suman Saurabh, S. Srikanth, K.H. Naveen, T. Prasanna, Neeti Rustagi, Prem Prakash Sharma

**Affiliations:** 1 Department of Community Medicine & Family Medicine, All India Institute of Medical Sciences, Jodhpur, Rajasthan India; 2 Veer Chandra Singh Garhwali Govt. Institute of Medical Science & Research, Srinagar, Uttarakhand India; 3 Department of Community & Family Medicine, All India Institute of Medical Sciences, Bhopal, India

**Keywords:** NCD, Diabetes, Hypertension, CVD, IDRS, CBAC, BMI, Waist Hip Ratio

## Abstract

**Background:**

The aim of the present study was to recalibrate the effectiveness of Indian Diabetes Risk Score (IDRS) and Community-Based Assessment Checklist (CBAC) by opportunistic screening of Diabetes Mellitus (DM) and Hypertension (HT) among the people attending health centres, and estimating the risk of fatal and non-fatal Cardio-Vascular Diseases (CVDs) among them using WHO/ISH charts.

**Methods:**

All the people aged ≥ 30 years attending the health centers were screened for DM and HT. Weight, height, waist circumference, and hip circumferences were measured, and BMI and Waist-Hip Ratio (WHR) were calculated. Risk categorization of all participants was done using IDRS, CBAC, and WHO/ISH risk prediction charts. Individuals diagnosed with DM or HT were started on treatment. The data was recorded using Epicollect5 and was analyzed using SPSS v.23 and MedCalc v.19.8. ROC curves were plotted for DM and HT with the IDRS, CBAC score, and anthropometric parameters. Sensitivity (SN), specificity (SP), Positive Predictive Value (PPV), Negative Predictive Value (NPV), Accuracy and Youden’s index were calculated for different cut-offs of IDRS and CBAC scores.

**Results:**

A total of 942 participants were included for the screening, out of them, 9.2% (95% CI: 7.45–11.31) were diagnosed with DM for the first time. Hypertension was detected among 25.7% (95% CI: 22.9–28.5) of the participants. A total of 447 (47.3%) participants were found with IDRS score ≥ 60, and 276 (29.3%) with CBAC score > 4. As much as 26.1% were at moderate to higher risk (≥ 10%) of developing CVDs. Area Under the Curve (AUC) for IDRS in predicting DM was 0.64 (0.58–0.70), with 67.1% SN and 55.2% SP (Youden’s Index 0.22). While the AUC for CBAC was 0.59 (0.53–0.65). For hypertension both the AUCs were 0.66 (0.62–0.71) and 0.63 (0.59–0.67), respectively.

**Conclusions:**

IDRS was found to have the maximum AUC and sensitivity thereby demonstrating its usefulness as compared to other tools for screening of both diabetes and hypertension. It thus has the potential to expose the hidden NCD iceberg. Hence, we propose IDRS as a useful tool in screening of Diabetes and Hypertension in rural India.

**Supplementary Information:**

The online version contains supplementary material available at 10.1186/s12889-022-12783-z.

## Background

India has been described as the diabetic capital of the world. It is home to 19% of the total world’s people living with diabetes. In terms of absolute numbers, it is expected to rise to 69.9 million by 2025 and 80 million by 2030 [1]. Unfortunately, these figures are just the tip of the iceberg, as more than half of the people with diabetes in the country remain undiagnosed [2]. This increases the risk of developing diabetic complications. According to the ICMR-INDIAB (ICMR-India Diabetes) study, the ratio of undiagnosed to diagnosed diabetes (DM) is higher in rural areas as compared to urban areas [3]. This emphasises the significance of community-wide awareness, screening, and early intervention.

Under the National Programme for Prevention & Control of Cancer, Diabetes, Cardiovascular Diseases & Stroke (NPCDCS), the Government of India has already started opportunistic screening of major Non-Communicable Diseases (NCDs). The Indian Diabetes Risk Score (IDRS), which was developed by Madras Diabetes Research Foundation (MDRF), Chennai, is based on two modifiable (waist circumference and physical inactivity) and two non-modifiable (age and family history of diabetes) known risk factors of DM. It has proven to be a simple and cost-effective method of predicting yet to be diagnosed diabetes. It possesses the sensitivity of 72.5% and specificity of 60.1% [4]. Several other studies [5, 6] have also validated the sensitivity, specificity, and accuracy of IDRS.

In India, a Community Based Assessment Checklist (CBAC) is used under NPCDCS to identify high-risk people for Non-Communicable Diseases (NCDs) [7]. It is based on four modifiable (smoking, alcohol, waist circumference, and physical inactivity) and two non-modifiable (age and family history of blood pressure/DM/heart disease) known risk factors for NCDs. This checklist is extensively used by grassroot health workers, although there is little research to back it up.

Nearly half of all NCD-related deaths are caused by Cardio-Vascular Diseases (CVDs) [8]. Risk factor identification and reduction has been regularly demonstrated in the scientific literature to be a better method for lowering CVD mortality than diagnosis and treatment (secondary prevention) [9–11]. The World Health Organization (WHO) and the International Society of Hypertension (ISH) have developed risk prediction charts to estimate the total CVD risk over a 10-year period [12]. These are basic color-coded charts that front-line health professionals can use in the field.

To investigate the ever increasing burden of NCDs, it is critical to identify the hidden spectrum using simple, non-invasive screening tools designed for the general population. In a large country like India, various available tools (IDRS, CBAC, and WHO/ISH) should be validated according to different geographical locations. Therefore, the present study was conducted to recalibrate the effectiveness of IDRS and CBAC by opportunistic screening for DM and hypertension (HT) among the people attending health centers, and estimating the risk of fatal and non-fatal CVDs among them using WHO/ISH charts.

## Methods

This hospital-based cross-sectional study was carried out from January 2019 to December 2019 in the three Rural Health Training Centres (RHTCs) of the Department of Community Medicine and Family Medicine of an apex medical institute in Jodhpur, India. Of these three centres, one is Community Health Centre (CHC) and the other two are Primary Health Centres (PHCs). In India, the CHCs and PHCs are created based on population norms. The catchment area of one CHC serves approximately 1.2 lakh population, while each PHC serves approximately 30,000 populations. Thus the catchment areas of all the three health centres included in this study serve approximately one lakh sixty thousand population. All the people aged ≥ 30 years who visited these health centres for various diseases (including those accompanying the patients) were included in the study. Patients with already diagnosed diabetes mellitus type 1 or type 2 or hypertension, pregnant females, and those not willing to give consent were all excluded from the study.

Random Capillary Blood Glucose (RCBG) was used to screen for DM. Those with a blood glucose of more than 140 mg/dl were sensitized about the risk of diabetes and were asked to attend the health center the next day for Fasting Plasma Glucose (FPG) after maintaining at least 8 h of fasting. RCBG and FPG were assessed using a glucometer by pricking the pulp of the left ring finger with a sterile lancet after applying the spirit swab. DM was diagnosed when a participant had an FPG level of ≥ 126 mg/dl. Prediabetes was diagnosed if the FPG value was 110–125 mg/dl [13].

Blood Pressure (BP) was measured using a digital blood pressure monitor. It was ensured that the participants had not consumed caffeine, tobacco, or exercised in the last 30 min, and that the patient has sat still for at least 5 min. To assess BP, the patients were asked to sit with their back straight, feet flat, and legs uncrossed with their arm on a flat surface at the heart level. Two blood pressure reading were taken at 1-min interval using a cuff of appropriate size. The average of the two readings was taken as the blood pressure reading of the participant. Hypertension was diagnosed using the Joint National Committee (JNC) VIII criteria [14]. Those with systolic BP ≥ 140 mmHg and/or diastolic ≥ 90 mmHg were diagnosed as person having hypertension.

A pre-validated and pretested (through a pilot study on 30 participants in a non-study area) interview schedule was administered to collect the information about sociodemographic details and risk factors for diabetes and hypertension. Weight of all the participants was measured with a digital weighing machine, height with a stadiometer, and waist and hip circumferences with a measuring tape. Risk assessment of obesity was done by calculating BMI and Waist Hip Ratio (WHR). Asian cut-off for BMI was adopted, with categories: < 18.5 underweight, 18.5–22.9 (normal), 23–24.9 (overweight), and ≥ 25 (obese) [15].

Risk categorization of all participants was done using three instruments; IDRS, CBAC, and WHO/ISH risk prediction charts (for 10-year risk of fatal or non-fatal cardiovascular event). In IDRS the total score spans from 0 to 100, and the participants were categorized as low risk (< 30), moderate risk (30–50), and high risk (≥ 60) based on their scores [16]. In CBAC, a score above 4 indicates a higher risk of developing NCDs [17]. WHO/ISH chart for SEAR (D) of WHO epidemiological sub-region was used to estimate the 10-year risk of CVD of all participants. The chart is used to categorise individuals into one of 5 categories: less than 10%; 10 to < 20%; 20 to < 30%; 30 to < 40%; and ≥ 40%.

All the data was recorded using Epicollect5, developed by Imperial College, London. Data was analyzed using SPSS v.23 and MedCalc v.19.8. Tables were used to display descriptive statistics. ROC curves were plotted for diabetes and hypertension with the dependent variables such as NCD risk scoring (IDRS, CBAC) and anthropometric parameters. Sensitivity (SN), specificity (SP), Positive Predictive Value (PPV), Negative Predictive Value (NPV), Accuracy and Youden’s index were calculated for different cut-offs of IDRS and CBAC scores.

The study was approved by Institutional Ethics Committee. Informed consent was obtained from all participants. All participants were explained about the risk factors for NCDs. People newly diagnosed with diabetes or hypertension were started on treatment and referred to tertiary care hospital to screen for complications. These patients were provided detailed information regarding their disease, its complication, and the lifestyle and dietary modification that were required. Participants having pre-diabetes and pre-hypertension were explained about their risk to develop diabetes or hypertension and were recommended to have regular check-ups as per the guidelines of NPCDCS program.

## Results

A total of 984 people were eligible to take part in the study, and 942 of them consented to undergo the screening process (non-consent rate; 4.2%). Socio-demographic, anthropometric and risk-related variables of the participants are depicted in supplementary table 1. Of these 942 participants, 223 (23.7%) were identified as screen-positives for diabetes and were invited to undergo FPG on next day. Out of these, 200 responded (response rate 89.6%). After excluding non-responders, the proportions of participants with prediabetes and DM were 6.42% (95% CI: 4.92–8.20) & 9.2% (95% CI: 7.45–11.31), respectively. All the participants (942) were screened for HT, and 25.7% (95% CI: 22.9–28.5) of them were diagnosed with HT.

A total of 447 (47.3%) study participants had a high risk of developing DM (IDRS score ≥ 60). According to CBAC score, 276 (29.3%) study participants were categorized as high-risk for NCDs (CBAC score > 4). As much as 26.1% of the participants were at moderate to higher risk (≥ 10%) of developing CVDs as per WHO’s risk prediction status (Table 1).

**Table 1:** Risk prediction of participants according to different scores (*n* = 942)

Variable	Category	*n* (%)
IDRS	< 30	54 (5.7)
50 (IQR:40–60)	30–50	442 (46.9)
	≥ 60	446 (47.3)
CBAC	< 4	666 (70.7)
4 (IQR: 3–5)	≥ 4	276 (29.3)
WHO RISK category	< 10%	696 (73.9)
	10% to < 20%	184 (19.5)
	20% to < 30%	33 (3.5)
	30% to < 40%	12 (1.3)
	≥ 40%	17 (1.8)

**Fig. 1:**
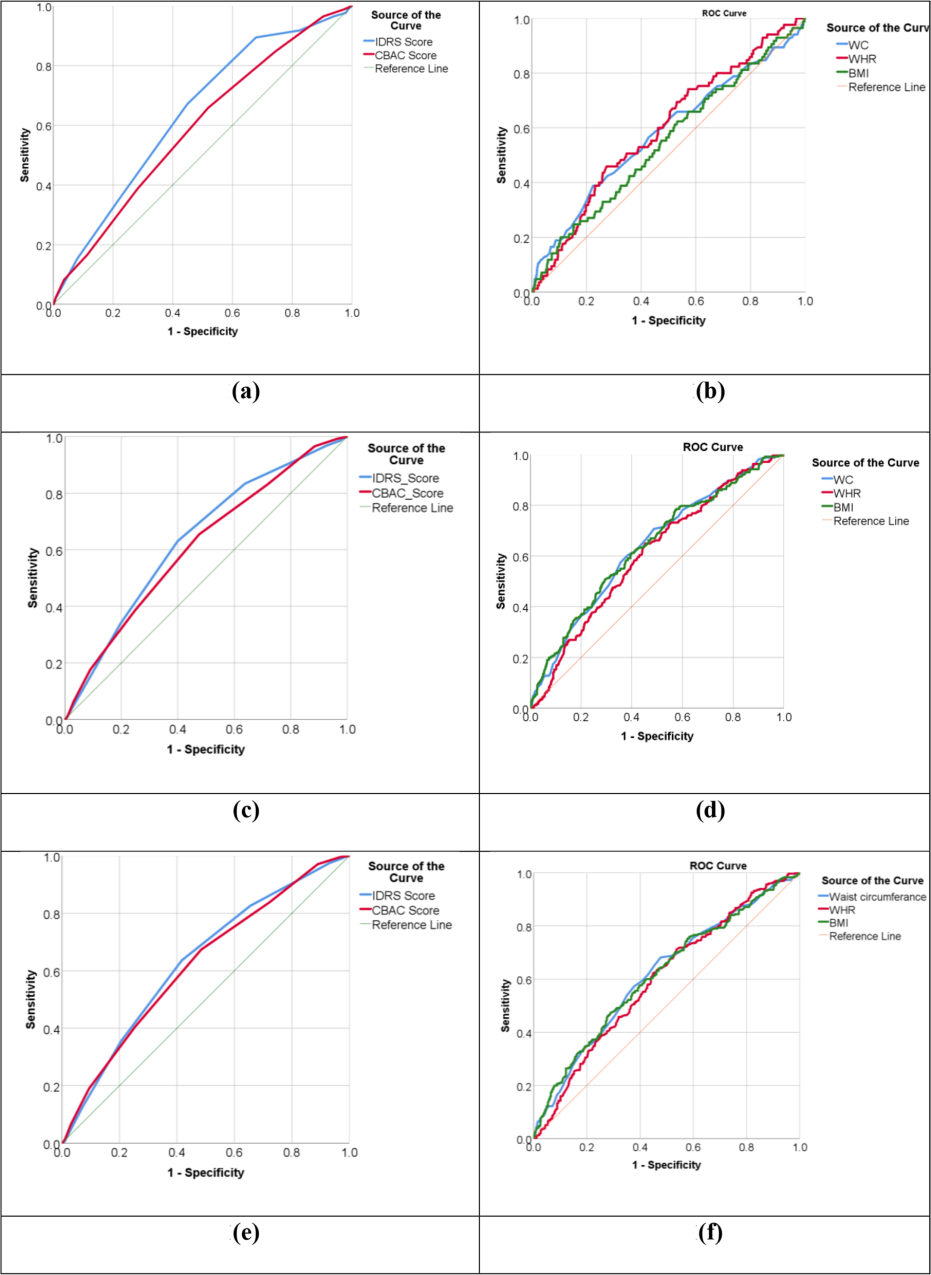
ROC curves; **a** Diabetes with IDRS and CBAC score, **b** Diabetes with anthropometric variables, **c** Hypertension with IDRS and CBAC score, **d** Hypertension with anthropometric variables, **e** Any NCD (HT or DM) with IDRS and CBAC score, **f** Any NCD (HT or DM) with anthropometric variables

Cut-off points of IDRS, CBAC scores and anthropometric parameters (WC, WHR and BMI) were estimated using ROC curves for DM, HT and any NCD (DM and/or HT) (Fig. 1).

All the variables included in the study like IDRS, CBAC, WC, WHR, and BMI had a statistically significant accuracy in determining any NCD. However, BMI was not found to be a statistically significant predictor of Diabetes Mellitus. The highest area under the curve was observed for IDRS for Diabetes [0.64 (0.58–0.70)], Hypertension [0.66 (0.62–0.71)] or for any NCD [0.64 (0.60–0.67)] (Table 2 and Fig. 1).

**Table 2:** Area Under the Curve (AUC) and level of significance for ROC curves

Variables	Diabetes		Hypertension		Any NCD (HT or DM)	
	**AUC (95% CI)**	**Sig**	**AUC (95% CI)**	**Sig**	**AUC (95% CI)**	**Sig**
IDRS	0.64 (0.58–0.70)	< 0.001	0.66 (0.62–0.71)	< 0.001	0.64 (0.60–0.67)	< 0.001
CBAC	0.59 (0.53–0.65)	0.006	0.63 (0.59–0.67)	< 0.001	0.61 (0.57–0.65)	< 0.001
WC	0.58 (0.51–0.65)	0.016	0.64 (0.60–0.68)	< 0.001	0.62 (0.58–0.66)	< 0.001
WHR	0.59 (0.53–0.66)	0.004	0.61 (0.57–0.65)	< 0.001	0.60 (0.56–0.64)	< 0.001
BMI	0.55 (0.48–0.61)	0.151	0.64 (0.60–0.68)	< 0.001	0.62 (0.58–0.66)	< 0.001

Tables 3, 4 and 5 provides the sensitivity, specificity, PPV, NPV and accuracy of different cut-offs for IDRS and CBAC for diagnosis of DM, HT and any NCD. An IDRS value ≥ 50 and CBAC value of ≥ 3 had the optimum sensitivity (89.4% and 84.7%, respectively) for determining diabetes. At the same cutoff values both the scores (IDRS and CBAC) were able to predict hypertension with the sensitivity of 82.6% and 83.9%, respectively.

**Table 3:** Sensitivity, Specificity, PPV, NPV, Accuracy and Youden’s index of IDRS and CBAC to diagnose diabetes

Score	Screen Positive	SN (%)	SP (%)	PPV (%)	NPV (%)	Accuracy (%)	Youden’s index
**IDRS**							
≥ 10	98.7	98.8	1.3	9.3	91.7	10.3	0.00
≥ 20	98.0	97.6	2.0	9.2	89.5	10.9	0.00
≥ 30	94.3	96.5	6.1	9.5	94.4	14.5	0.03
≥ 40	83.5	91.8	17.5	10.2	95.4	24.4	0.09
≥ 50	70.0	89.4	32.1	11.8	96.8	37.4	0.22
≥ 60	47.3	67.1	55.2	13.2	94.3	56.3	0.22
≥ 70	24.4	36.5	77.1	14.0	92.3	73.3	0.14
≥ 80	8.7	15.3	92.0	16.3	91.4	84.9	0.07
≥ 90	0.4	1.2	99.6	25.0	90.8	90.5	0.01
≥ 100	0.1	0.0	99.9	0.0	90.7	90.6	0.00
**CBAC**							
≥ 1	97.6	98.8	2.6	9.4	95.7	11.5	0.01
≥ 2	91.1	96.5	9.6	9.8	96.4	17.6	0.06
≥ 3	75.3	84.7	25.7	10.4	94.3	31.1	0.10
≥ 4	53.3	65.9	48.1	11.5	93.3	49.7	0.14
≥ 5	29.3	38.8	71.7	12.3	92.0	68.7	0.11
≥ 6	11.9	16.5	88.7	13.0	91.2	82.0	0.05
≥ 7	4.0	8.2	96.4	18.9	91.2	88.2	0.05
≥ 8	1.1	2.4	99.2	22.2	90.9	90.2	0.02
≥ 9	0.1	0.0	99.9	0.0	90.7	90.6	0.00

**Table 4:** Sensitivity, Specificity, PPV, NPV, Accuracy and Youden’s index of IDRS and CBAC to diagnose hypertension

Score	SN (%)	SP (%)	PPV (%)	NPV (%)	Accuracy (%)	Youden’s index
**IDRS**						
≥ 10	99.6	1.6	25.9	91.7	26.8	0.01
≥ 20	99.2	2.4	26.0	89.5	27.3	0.02
≥ 30	97.5	6.9	26.6	88.9	30.1	0.04
≥ 40	90.9	19.0	28.0	85.8	37.5	0.10
≥ 50	82.6	34.4	30.3	85.2	46.8	0.17
≥ 60	63.6	58.3	34.5	82.3	59.7	0.22
≥ 70	35.5	79.4	37.4	78.1	68.2	0.15
≥ 80	12.8	92.7	37.8	75.5	72.2	0.06
≥ 90	0.0	99.4	0.0	74.2	73.9	-0.01
≥ 100	0.0	99.9	0.0	74.3	74.2	0.00
**CBAC**						
≥ 1	99.6	3.1	26.2	95.7	27.9	0.03
≥ 2	97.1	11.0	27.4	91.7	33.1	0.08
≥ 3	83.9	27.7	28.6	83.3	42.1	0.12
≥ 4	67.4	51.6	32.5	82.0	55.6	0.19
≥ 5	40.5	74.6	35.5	78.4	65.8	0.15
≥ 6	19.0	90.6	41.1	76.4	72.2	0.10
≥ 7	6.6	96.9	42.1	75.0	73.7	0.04
≥ 8	1.2	99.0	30.0	74.4	73.9	0.00
≥ 9	0.0	99.9	0.0	74.3	74.2	0.00

**Table 5:** Sensitivity, Specificity, PPV, NPV, Accuracy and Youden’s index of IDRS and CBAC to diagnose any NCD (diabetes and/or hypertension)

Score	SN (%)	SP (%)	PPV (%)	NPV (%)	Accuracy (%)	Youden’s index
**IDRS**						
≥ 10	99.3	1.6	32.0	83.3	32.7	0.01
≥ 20	98.6	2.4	32.0	78.9	33.0	0.01
≥ 30	96.9	7.1	32.7	83.3	35.7	0.04
≥ 40	90.8	19.9	34.6	82.4	42.5	0.11
≥ 50	83.4	36.2	37.8	82.4	51.2	0.20
≥ 60	63.1	60.0	42.4	77.7	61.0	0.23
≥ 70	33.9	80.3	44.4	72.3	65.5	0.14
≥ 80	11.9	92.9	43.8	69.3	67.1	0.05
≥ 90	0.3	99.5	25.0	68.2	68.0	0.00
≥ 100	0.0	99.8	0.0	68.2	68.1	0.00
**CBAC**						
≥ 1	99.3	3.3	32.4	91.3	33.8	0.03
≥ 2	96.6	11.5	33.7	88.0	38.6	0.08
≥ 3	83.1	28.3	35.1	78.2	45.7	0.11
≥ 4	65.4	52.4	39.1	76.5	56.6	0.18
≥ 5	38.6	75.0	41.9	72.4	63.5	0.14
≥ 6	17.6	91.0	47.7	70.3	67.7	0.09
≥ 7	6.1	97.0	48.6	68.9	68.1	0.03
≥ 8	1.0	99.1	33.3	68.2	67.9	0.00
≥ 9	0.0	99.8	0.0	68.2	68.1	0.00

## Discussion

In this study, health facility-based opportunistic screening was conducted for DM and HT among rural people of Western Rajasthan to determine the proportion of newly diagnosed cases. The ten-year CVD risk of the participants was calculated using WHO’s risk prediction charts. The prediction capacity of the IDRS and CBAC scores was recalibrated to screen for DM and HT, and the appropriate cut-offs were identified.

In our study, AUC for IDRS score in predicting DM was 0.64 (95%CI: 0.58–0.70), with 89.4% sensitivity and 32.1% specificity at the cut-off of ≥ 50, and with 67.1% sensitivity and 55.2% specificity at the cut-off of ≥ 60. Both the cut-offs were having the same Youden’s Index (0.22). A large population-based study in the Indian urban and rural population has shown 72.5% sensitivity and 60.1% specificity for the IDRS score cut-off ≥ 60 [4]. Another study conducted in an urban slum in India has depicted the sensitivity of 95.12% and specificity of 28.9% at ≥ 60 cut-off score of IDRS [18]. In the study by Bhadoria et al. among the adult population in central India recommended the IDRS cut-off value of 40 with a sensitivity, specificity, and Youden index of 60.4%, 70.7%, and 0.31, respectively [19].

Though IDRS score is traditionally used for screening for Diabetes, in the present study we demonstrated its application in screening for hypertension (AUC: 0.66, 95% CI:0.62–0.71) with 82.6% sensitivity and 34.4% specificity at the cut-off of ≥ 50 (Youden’s Index, 0.17), and 63.6% sensitivity and 58.3% specificity at the cut-off of ≥ 60 (Youden’s Index, 0.22). There is a paucity of scientific evidences exploring the prediction capacity of IDRS for other NCD. The association of IDRS with hypertension has been reported in the Indian setting by Garg et al. [20].

The findings of the present study depict that, the CBAC score, which is widely used by the frontline health worker in India to screen for NCDs, poorly predicted DM (AUC:0.59, 95% CI:0.53–0.65), hypertension (AUC:0.63, 95% CI:0.59–0.67) as well as any NCD (AUC:0.61, 95% CI:0.57–0.65) as compared to IDRS (AUC for any NCD:0.64, 95%CI:0.60–0.67). There is a dearth of scientific evidences exploring the prediction capacity of CBAC, thus making comparison difficult. The CBAC checklist includes additional questions on tobacco and alcohol use as compared to IDRS. The greater predictive accuracy of IDRS could be attributed to relatively higher importance given to age, family history, physical activity and central obesity as compared to the role of tobacco and alcohol use in the causation of NCDs. Further, both the amount and frequency of alcohol and tobacco use would be important, which is not captured in CBAC. Nevertheless, tobacco use has an important role when predicting the risk of cardiovascular events and mortality.

Near about one-fourth of the participants were at moderate to a higher risk of developing CVDs as per WHO’s risk prediction charts. The application of WHO’s CVD risk prediction charts for the Indian population has been studied by many authors in different parts of the country. The finding of the present study is in accordance with the findings reported by Deori T.J. et.al. in the rural population of Central India (23.1%) and Raghu et. Al. in the rural part of South India (25.2%) [21, 22]. Contrary to this, Ghorpade et.al. have shown only 17% of the participants at moderate to high risk for the occurrence of cardiovascular events by using WHO/ISH risk prediction charts in a rural population of South India [23].

This study further supports the role of anthropometric indicators in predicting NCDs. WC and WHR were significantly predicting DM, while hypertension was predicted by WC, WHR and BMI. A strong association of BMI with hypertension has been demonstrated in the scientific world [24, 25]. A meta-analysis of Indian studies evidenced a statistically significant association of obesity with type 2 DM (pooled OR = 1.14; 95%CI: 1.043 to 1.237) and hypertension (pooled OR = 3.820; 95%CI: 3.392 to 4.248) [26].

In the present study, we did not explore about the symptoms of diabetes and hypertension among the participants. Though it is not affecting the final objectives of the study, yet it may cause bias to the performance of the studied tool. This may be considered as a limitation of the study.

## Conclusion

The current study provides scientific evidence by recalibrating IDRS and CBAC as instruments for screening of DM and HT in a rural area of Western India. IDRS was found to have the maximum AUC and sensitivity thereby demonstrating its usefulness as compared to other tools for screening of both diabetes and hypertension. It thus has the potential to expose the hidden NCD iceberg. Hence, we propose IDRS as a useful tool in screening of Diabetes and Hypertension in rural India. Rather than avoiding false positives, this is the time to expose the hidden section of the iceberg of NCDs by having high sensitivity of non-invasive instruments (like IDRS). As a result of the findings of this study, we propose a cut-off value of 50 for the IDRS to screen for diabetes in the rural population.

## Supplementary Information

**Additional file 1: Table 1.** Sociodemographic characteristics of the study population (*n*=942).

## Data Availability

The datasets used and/or analysed during the current study are available from the corresponding author on reasonable request.

## Abbreviations

AUC: Area Under the Curve
BMI: Body Mass Index
BP: Blood Pressure
CBAC: Community Based Assessment Checklist
CHC: Community Health Centre
CI: Confidence Interval
CVDs: Cardio-Vascular Diseases
DM: Diabetes Mellitus
FPG: Fasting Plasma Glucose
HT: Hypertension
IDRS: Indian Diabetic Risk Score
ISH: International Society of Hypertension
JNC: Joint National Committee
MDRF: Madras Diabetes Research Foundation
NCDs: Non-Communicable Diseases
NPV: Negative Predictive Value
PHC: Primary Health Centre
PPV: Positive Predictive Value
RCBG: Random Capillary Blood Glucose
ROC: Receiver Operating Characteristic Curve
SN: Sensitivity
SP: Specificity
WC: Waist Circumference
WHO: World Health Organization
WHR: Waist Hip Ratio
